# Laser Heating Study of the High-Temperature Interactions in Nanograined Uranium Carbides

**DOI:** 10.3390/ma14195568

**Published:** 2021-09-25

**Authors:** Sanjib Chowdhury, Dario Manara, Oliver Dieste-Blanco, Davide Robba, António Pereira Gonçalves

**Affiliations:** 1 C^2^TN, DECN, Instituto Superior Técnico, Campus Tecnológico e Nuclear, Universidade de Lisboa, Estrada Nacional 10, 2695-066 Bobadela LRS, Portugal; sanjibbua@gmail.com; 2 Joint Research Centre (JRC), European Commission, Directorate of Nuclear Safety and Security, P.O. Box 2340, D-76125 Karlsruhe, Germany; Dario.MANARA@ec.europa.eu (D.M.); oliver.dieste@gmail.com (O.D.-B.); davide.robba@ec.europa.eu (D.R.)

**Keywords:** uranium carbide, nanomaterials, nuclear materials, laser heating, spallation targets

## Abstract

Nanograined nuclear materials are expected to have a better performance as spallation targets and nuclear fuels than conventional materials, but many basic properties of these materials are still unknown. The present work aims to contribute to their better understanding by studying the effect of grain size on the melting and solid–solid transitions of nanograined UC_2−y_. We laser-heated 4 nm–10 nm grain size samples with UC_2−y_ as the main phase (but containing graphite and UO_2_ as impurities) under inert gas to temperatures above 3000 K, and their behavior was studied by thermal radiance spectroscopy. The UC_2−y_ solidification point (2713(30) K) and α-UC_2_ to β-UC_2_ solid–solid transition temperature (2038(10) K) were observed to remain unchanged when compared to bulk crystalline materials with micrometer grain sizes. After melting, the composite grain size persisted at the nanoscale, from around 10 nm to 20 nm, pointing to an effective role of carbon in preventing the rapid diffusion of uranium and grain growth.

## 1. Introduction

The Isotope Separator On-Line (ISOL) technique allows the production of a wide variety of radioisotopes that can be used in many applications, such as nuclear physics, astrophysics, solid-state sciences, and medicine [1,2]. In this method, a high-energy beam of atomic or subatomic particles hits a nuclear spallation target. The isotopes are formed by fragmentation, fission, and spallation nuclear reactions inside the target material. They then diffuse, effuse, vaporize, or sublimate; pass through an ionizer cavity; and are electrostatically accelerated and separated. The total process time must be less than the half-life of the isotopes in order to be effective and useful. Therefore, high temperatures are usually needed to ensure the rapid diffusion, effusion, and vaporization or sublimation of isotopes [3].

Uranium carbide-based materials, especially UC_x_ composites, are the world reference material for ISOL spallation targets [4]. Moreover, they have a good potential to be also used as nuclear fuels of advanced generation IV reactors, particularly the gas-cooled fast reactors [5]. Three phases exist in the U–C binary system at atmospheric pressure: UC and UC_2−y_, which are stable up to melting (>2700 K); and U_2_C_3_, which decomposes peritectically into UC_1+x_ and UC_2−y_ at ~2100 K [6]. The first two (UC_1+x_ and UC_2−y_) are useful for the above-mentioned applications, their ability for these purposes relying on their specific chemical and physical properties, such as high-temperature stability, lack of phase transformations at practical temperatures, high fissile metal densities, high melting points, and high thermal conductivities [7,8]. As an example, uranium carbide nuclear fuels are expected to show an improved performance under severe loss-of-coolant accidents (LOCAs) [9] due to their high thermal conductivity, which leads to a reduction in the thermal energy stored at the fuel. They also have a higher uranium density when compared to UO_2_, which makes it possible to decrease the initial fuel enrichment in fissile nuclei. Concerning the granulometry of these materials, recent investigations made on different specimens pointed to a beneficial influence of the grain size decrease to the nanoscale in both applications (nuclear fuels and spallation targets) [10,11,12,13], which is also expected to be the case of uranium carbides. However, many basic properties of such nanostructured materials, fundamental for their assignment as nuclear fuels and high-performance targets, are unknown or incompletely studied. In particular, the grain size reduction to nanometric dimensions can have a dramatic effect on properties like the melting point, which can decrease by hundreds of degrees [14]. This decrease is mainly ascribed to the relevant role played by surface tension, in line with the increased surface-to-volume ratio, which changes the general thermodynamic and thermal properties of the material [14].

This study focusses on the high-temperature behavior of nanograined uranium carbides to be employed as ISOL spallation targets and, potentially, as advanced nuclear fuels. Such materials are likely to be heated to high temperatures during their possible operation, for example, by proton irradiation, if used as a spallation target, or by nuclear reactions, if used as a nuclear fuel. It is therefore essential to assess their response to high-temperature conditions and, in particular, the phase transitions they undergo. Experimental data of this kind have been published for bulk uranium carbides [6,15,16] but are absent for nanostructured carbides. Here we present the investigation of the solid–solid and liquid–solid transitions occurring above 2000 K in nanograined UC_2−y_, containing graphite and UO_2_ as secondary phases, using a laser heating technique coupled with fast and multi-wavelength optical spectro-pyrometry. This study is intended to evaluate the influence of the nanocrystalline grain size on the melting temperature of such materials, when compared with micrometric ones. The characterization of the structure, microstructure, and composition of the samples was performed before and after heating by the use of powder X-ray diffraction, Raman spectroscopy, and scanning and transmission electron microscopies, coupled with energy-dispersive X-ray spectroscopy.

## 2. Materials and Methods

### 2.1. Materials Preparation

UC_2−y_ materials with nanometric grain size were prepared by (i) electrospinning a solution containing uranyl acetate and cellulose acetate with a 15% concentration (*w*/*v*) of U/C = 1/4, followed by (ii) rapid decomposition under an argon gas flow atmosphere (by inserting the sample inside a furnace pre-heated at 550 °C for 1 h) and (iii) carboreduction under vacuum at 1800 °C for 2 h in a radioinduction furnace, as described in [17]. The prepared material was mixed with a very small proportion of zirconia glue (ZrO_2_ suspended in ethanol), which was kept at 80 °C overnight in order to evaporate the solvent. The mixture was shaped to cylinders of 5 mm diameter and 2 mm thickness by cold pressing at 1 ton for two hours, and the pellets were used in the experiments.

### 2.2. Characterization

The morphology of the materials was studied by scanning electron microscopy (SEM) using a VEGA TESCAN^®^ SEM operated at 20–30 kV and equipped with energy dispersive spectroscopy (EDX) analysis systems (SAMx EDS SD-Detector) to evaluate the chemical composition of the phases. Transmission electron microscopy (TEM) images and selected area electron diffraction (SAED) patterns were taken on hand-crushed samples using a JEOL FX2100 (Tokyo, Japan) at 200 kV. The TEM samples were prepared by manually crushing tiny fragments of the UC_2−y_ materials in methanol. The suspension was decanted, and a droplet was subsequently deposited on a carbon-coated copper grid TEM sample holder. The SAED patterns were indexed using the SingleCrystal analysis software (CrystalMaker Software Ltd., Begbroke, UK).

Pre- and post-laser-heated samples were manually powdered in a mortar grinder, and the resulting powder was placed onto a low-noise Si single-crystal sample holder and studied by powder X-ray diffraction. The X-ray diffraction measurements were performed in a Bruker^®^ D8 Advance diffractometer (Cu Kα radiation) with Bragg–Brentano geometry. X-ray diffraction patterns were taken at operating conditions of 40 kV and 40 mA in the step-scanning mode for the 2θ 15–80° regions using the θ/2θ configuration, a step size of 0.04° and a counting time per step of 40.00 s. The theoretical powder patterns were simulated with the help of the Powder Cell program [18] and compared with the experimental patterns.

Raman spectra measurements were made using a Jobin-Yvon^®^ T64000 spectrometer in the single spectrograph configuration. The 647 nm line of a Kr+ Coherent^®^ laser was used as an excitation source, with a nominal power at the laser cavity of 100 mW. This wavelength and power were chosen in order to optimize the signal/noise ratio and reduce undesirable oxidation/burning effects on the sample surface. 

### 2.3. The Laser-Heating Method

At a given wavelength and temperature, a real body radiates only a fraction of the power emitted by an ideal black body at the same temperature. The emissivity of the body, *ε*, is the ratio between its radiant emittance and the radiant emittance of a black body at the same temperature under given conditions. In particular, the normal spectral emissivity, *ε_λ_*, expresses the emissivity at a given frequency and at a solid angle normal to the sample surface, conditions that can be more conveniently reproduced in a laboratory. *ε_λ_* takes values between 0 and 1, with 1 corresponding to the ideal black-body case, for which Planck’s law was derived. The sample normal spectral radiance, *L_ex_*, is linked to the sample surface temperature *T* through a modified Planck function [19]:

(1) $$L_{ex}=\frac{L_{\lambda }}{c_{1}}=\frac{1}{\lambda^{5}}\cdot \frac{\varepsilon_{\lambda }\left(T\right)}{e^{\frac{c_{2}}{\lambda \cdot T}}-1}$$

where *L_λ_* represents the radiative power, *ε_λ_* is the normal spectral emissivity, and *c*_1_ = 2.*h*.*c_o_*^2^ and *c*_2_ = *h.c_o_/k_B_* are the first and second radiation constants (*c_o_* is the speed of light in vacuum, *h* is Planck’s constant, and *k_B_* Boltzmann’s constant). The normal spectral emissivity of a material can be determined by making a non-linear fit of the thermal emission spectrum with Equation (1), where *T* and *ε_λ_* are the only two free parameters. This approach has been demonstrated to be acceptably accurate in refractory materials, like those usually present in nuclear power plants, for which the normal spectral emissivity can be assumed to be wavelength-independent (grey-body hypothesis) in a broad spectral range [20]. This grey-body assumption is not rigorously correct for actinide carbides. However, the normal spectral emissivity behavior of uranium carbides as a function of wavelength in the visible–near infrared spectrum was accurately determined in recent studies [6,15], providing the values needed for the present temperature determination.

**Figure 1:**
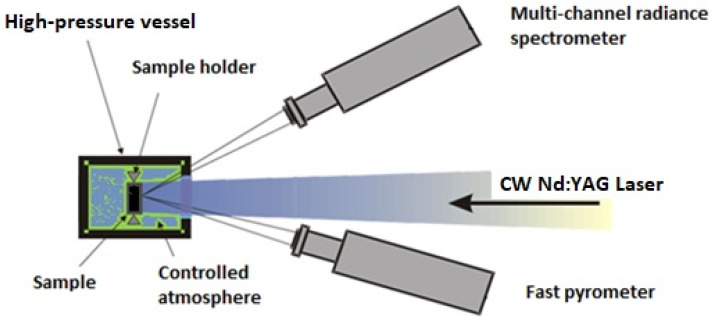
Schematic of the laser-heating set-up employed in this work.

The current investigation was made by employing the laser-heating method presented in [21,22] (Figure 1), with the temperatures being determined using radiation pyrometers (the only devices available to measure temperatures above 2500 K). The samples (pellets) were mounted in a pressurized steel vessel with 0.2–0.3 MPa argon in order to avoid oxidation. A 4.5 kW Nd:YAG continuous-wave laser (HLD4506, TRUMPF, Schramberg, Germany) that allows shots of different durations (from tens to hundreds of milliseconds) and power densities (300 W cm^−2^ to 3000 W cm^−2^) was used to heat a ~5 mm diameter circular area. The sample radiance, *L_ex_* (the electromagnetic radiation power density per unit surface, wavelength, and solid angle thermally emitted by the sample at a given temperature), was measured using radiation pyrometers, which were always set up with an architecture normal to the sample surface. Therefore, in the present work, *L_ex_* and *ε_λ_* always refer to the normal radiance and spectral emissivity, respectively. The temperature at the center of the heated area was measured using a fast (10 µs) 655 nm two-channel pyrometer calibrated against standard lamps up to 2500 K [21].

An additional, slower (1 ms) 256-channel radiance spectro-pyrometer operating between 515 and 980 nm was employed for the analysis of its optical properties. In this last pyrometer, the 649 nm channel was calibrated using the same procedure as for the fast pyrometer, while the remaining channels were calibrated against a graphite black body operating up to 3300 K. The standard lamps were in turn calibrated at 650 nm by one of the German standard reference institutions, the PTB (Physikalisch Technische Bundesanstalt)—the first one for the temperature range between 1100 and 1800 K, the second for between 1800 and 2500 K. The radiance temperature of the reference lamps was established with a ±0.5% accuracy as a function of the current flowing in the lamp filament. Calibration of the pyrometers was therefore obtained by focusing the pyrometer objective on a predefined point of the lamp filament and by recording the pyrometer’s output voltage corresponding to the detected lamp radiance at a given temperature. In this way, a voltage vs. radiance temperature curve was obtained for each pyrometer. The same procedure was carried out for the calibration of the multichannel spectro-pyrometer. In this latter case, however, a calibrated black-body source was employed instead of a standard lamp, as a broader spectral range needed to be covered (515–980 nm). The black-body source was heated by the Joule effect, and its temperature was directly measured using the single-channel pyrometer previously calibrated against the standard lamps. The maximum black-body temperature measured during the calibration procedure was 3000 K. By applying this procedure to the melting/solidification transitions of some secondary reference materials (such as tungsten and molybdenum [23]), an estimate of an approximately 20 K uncertainty at 2000 K was obtained. Based on that, an uncertainty of 1% on the measured temperature was estimated for the current measurements (more details about the pyrometer calibration and the current experimental technique are reported in [24]).

Sequences of four laser pulses with different power vs. time shapes were used in this study. The pulse durations varied between 200 and 400 ms, and a maximum power of 585 W was used, which led to maximum local temperatures of ~3200 K. Most of the heat delivered onto the sample diffused into the bulk due to the high thermal conductivity of UC_2−y_, but there was local heating of the sample surface, leading to its melting. Excessive thermal shocks were minimized by starting each series of pulses with a pre-heating pulse that stabilized the sample surface temperature around 1800 K for 200 ms. The sample was not allowed to cool below 1500 K between the pulses. Both the pulse power and duration were increased from one cycle to another to ensure better mechanical stability. Thermal analysis was performed on the temperature–time curves once the temperature of the laser-heated sample was correctly determined as a function of time. The digitized output thermograms (temperature vs. time curves) were plotted with a 1 ms resolution. The final thermal arrest temperatures were calculated by averaging the values obtained in each laser pulse.

The most significant uncertainty sources related to the laser heating and multi-channel pyrometry were combined according to the independent error propagation law (Equation (2)) and expanded to yield relative temperature uncertainty bands corresponding to 2 standard deviations (*k* = 2 coverage factor). These uncertainty components stem from our current temperature-scale definition *δT* (i.e., the uncertainty in the pyrometer calibration), the spectral emissivity assessment $\delta T_{\varepsilon_{\lambda }}$, and the experimental data scatter on the current phase transition (radiance) temperature data $\delta T_{\lambda_{m}}$, the latter being the main source of uncertainty [21]:

(2) $$\delta T_{m}=\sqrt{\delta T^{2}+\delta T_{\varepsilon_{\lambda }}^{2}+\delta T_{\lambda_{m}}^{2}}$$

The resulting cumulative uncertainty is on the order of ±20 K at 2000 K; thus, an uncertainty of ±1% was used for the values of temperature measured in this work.

## 3. Results and Discussion

**Figure 2:**
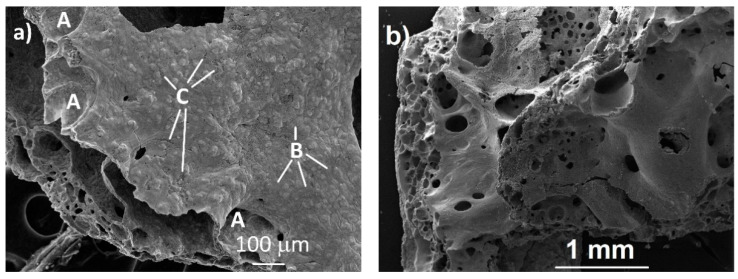
SEM micrographs of the (**a**) pre- and (**b**) post-laser-heated materials. A, B and C indicate the position of large holes, small grains, and pores, respectively.

SEM micrographs of the pre- and post-laser-heated materials are displayed in Figure 2.

**Figure 3:**
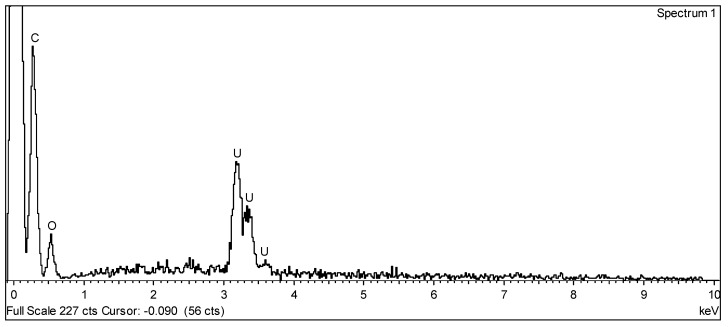
EDX spectrum of the UC_2−y_ material before laser heating, showing the presence of uranium, oxygen, and carbon.

The as-synthesized material in Figure 2a shows a surface with large holes (A), small grains (B), and pores (C). The aspect of the as-synthesized sample points to the partial melting of the electrospun material during the decomposition heat treatment before the pyrolysis. This agrees with the melting temperatures reported for the major constituent of these samples (i.e., cellulose acetate polymer; Tm = 240–300 °C), which is much lower than the 550 °C used to decompose the electrospun material. EDS analysis of as-synthesized samples shows the presence of uranium, oxygen, and carbon (Figure 3). 

The even unquantified presence of oxygen is a clear indication that the carbothermal reaction

**Figure 4:**
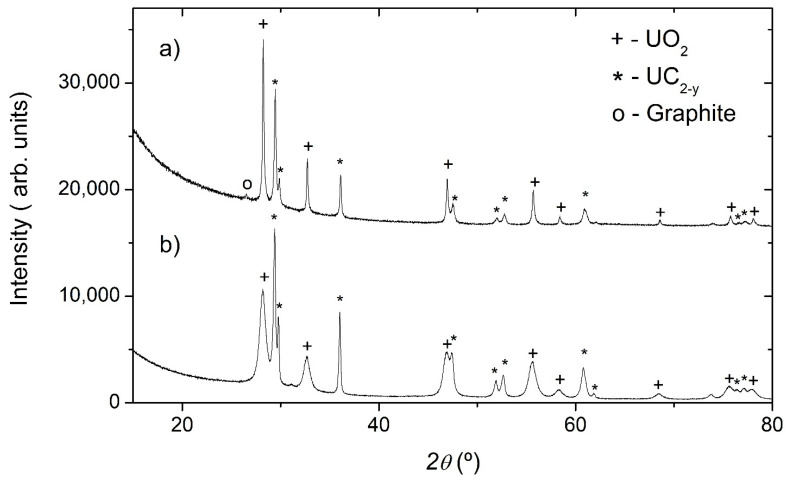
X-ray diffractograms of UC_2−y_ material (**a**) before and (**b**) after laser heating.

UO_2_ + 4C → UC_2−y_ + 2CO (3)

was not completed after the 2 h high-temperature heat treatment. X-ray diffraction measurements confirm this assumption, showing that UC_2−y_ and UO_2_ are the main constituents of the pre-laser-heated material (Figure 4a). 

**Figure 5:**
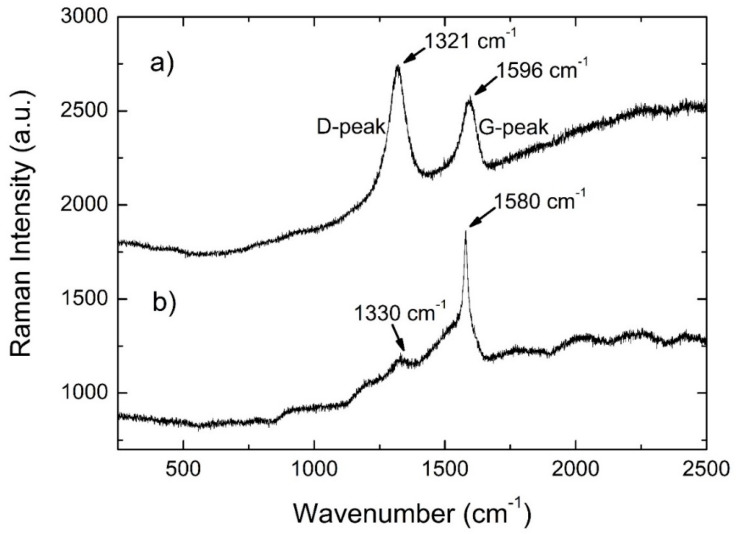
Raman spectra of the UC_2−y_ material (**a**) before and (**b**) after laser heating.

Although the initial molar ratio of U/C was 1/4, no significant diffracted signal from carbon (graphite) was observed, probably due not only to the light nature of this element but also to disorder, as suggested by the Raman analysis reported in Figure 5a. The refinement of the UO_2_ lattice parameter (*a* = 0.5469(1) nm) points to an oxide composition very close to the 1:2 stoichiometry [25], in contrast with previous results that indicate a hyperstoichiometric oxygen composition for these types of impurities in carbo-reduced materials [17]. On the other hand, the UC_2−y_ lattice parameters (*a* = 0.35149(2) nm, *c* = 0.5976(1) nm) are very close to the results obtained for UC_2−y_ fibers prepared using a similar procedure [17] and are in agreement with the published data [26]. The Raman spectrum of the as-fabricated material (Figure 5a) shows two broad bands centered at 1321 cm^−1^ and 1596 cm^−1^, which correspond to the D-peak of defective and disordered graphite and to the G-peaks of graphite, respectively [27]. The first is attributed to the breathing modes of the sp^2^ atoms ring, the second is due to bond stretching of all pairs of sp^2^ atoms, both in rings and chains. These results suggest the presence of a substantial amount of disordered graphite, as already suggested by the nonexistence of a significant diffracted signal from carbon on the diffractograms of the UC_2−y_ material before laser heating. No Raman active peaks exist in UC_2_. The lack of UO_2_ bands is most likely due to the excess carbon, which fully reacted with the oxide.

**Figure 6:**
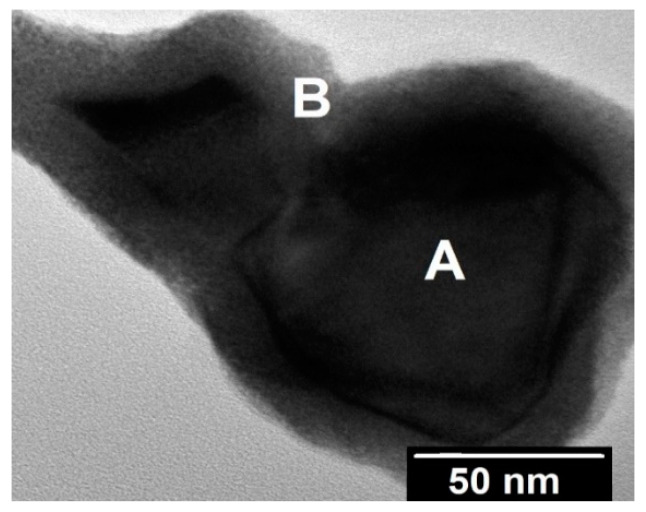
Bright-field TEM image with the difference in colors pointing to the presence of unreacted UO_2_ (A) surrounded by UC_2−y_ (B).

**Figure 7:**
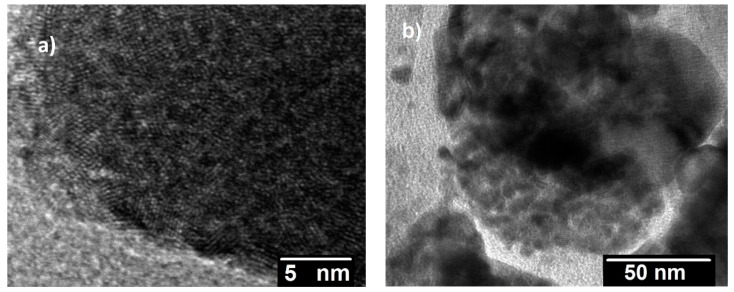
High-resolution TEM images of the UC_2−y_ material (**a**) before and (**b**) after laser heating.

**Figure 8:**
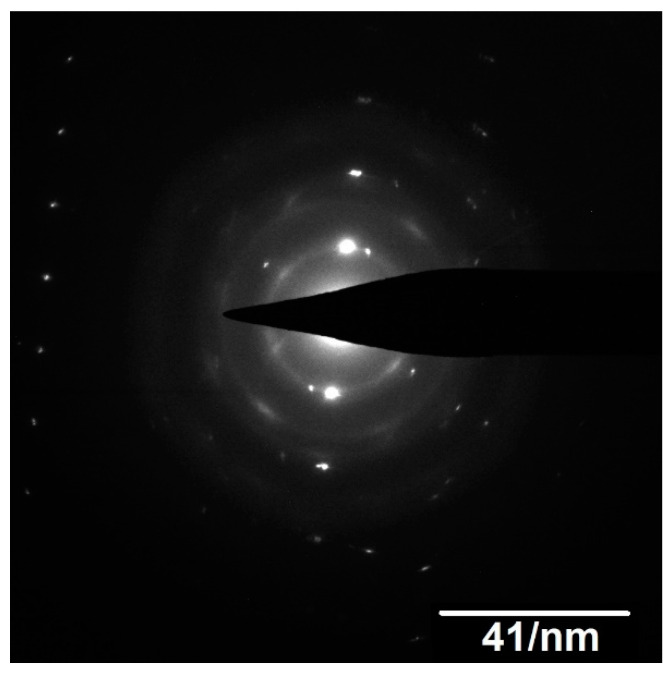
SAED pattern of the UC_2−y_ phase.

TEM observations support the partial carbo-thermal reaction of UO_2_, pointing to the presence of unreacted UO_2_ surrounded by UC_2−y_ (Figure 6). High-resolution TEM observations of the UC_2−y_ phase showed that the grain sizes were very small, ranging between 4 and 10 nm (Figure 7a), confirming this method is suitable for the preparation of nanograined UC_2−y_ materials. SAED measurements (Figure 8) corroborate the crystalline character of this phase, albeit also showing the presence of continuous amorphous rings, most probably reflecting the disorder at grain boundaries.

Laser heating is a powerful technique to study phase transitions at temperatures higher than 2000 K. However, in order to observe significant thermal arrests at the phase transition temperatures, it is not only necessary to exceed the transition temperatures themselves, but one must also ensure that a large enough material mass is heated beyond the phase transition, depending on the involved transition enthalpies. This can be done by irradiating the sample with laser pulses that are sufficiently intense and long, where the power and duration of laser pulses are determined by a trial-and-error procedure. 

**Figure 9:**
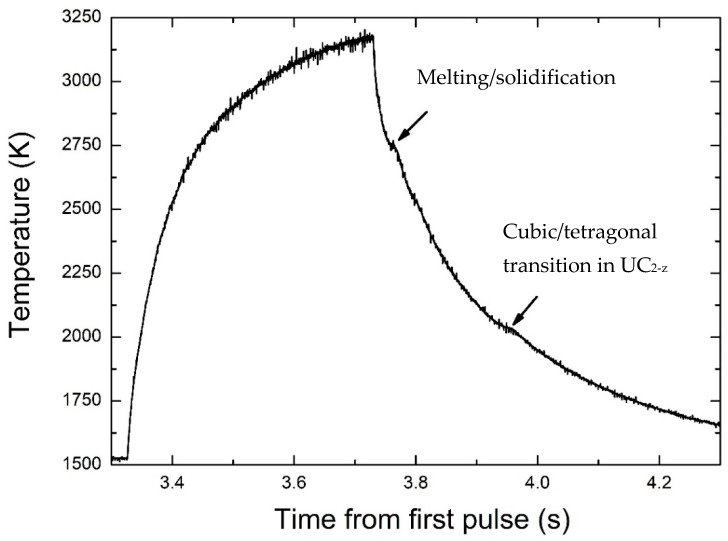
Thermogram of the UC_2−y_ material for a 585 W laser heating 400 ms shot. Arrows indicate the positions of the thermal arrests.

A typical thermogram recorded on a laser-heated UC_2−y_ sample is presented in Figure 9. It was obtained with a 400 ms laser pulse of 585 W. The thermogram, which shows the local sample surface temperature during the laser heating and cooling cycles, indicates a rapid increase to a maximum of about 3200 K, above the previously reported melting temperatures of the U–C binary phases [6]. After the end of the laser pulse, the temperature rapidly decreases, and two anomalies corresponding to thermal arrests due to phase transitions can be observed in the thermogram. It was proven, by repeating laser-heating cycles with different laser settings, that both temperature anomalies were independent of the laser pulse power and duration, within the experimental uncertainty. The lower-temperature anomaly observed at 2038(10) K matches well the previously described temperatures for the tetragonal to cubic α-UC_2−y_ → β-UC_2−y_ solid-state transition, which ranges from 2036 to 2080 K [6,15,28]. The higher-temperature thermal arrest was observed at 2713(30) K, which is slightly higher than the 2700 K reported for the β-UC_2−y_ melting temperature by Benz et al. [29] but lower than the 2737 K value more recently published [6]. These results indicate that the nanograined β-UC_2−y_ material had a solidification temperature similar to bulk crystalline materials with micrometer grain sizes. The observed temperature was technically a solidification temperature, which may differ from the melting point as the nanograins agglomerate in the liquid. Nonetheless, because of the short duration of the heating pule (less than 500 ms), grain agglomeration can be assumed to have happened to a very limited extent. This assumption calls for confirmation by post-melting material characterization.

The effect of laser treatment and repeated fusion was visible on the surface of the samples after laser heating. SEM observations show that the surface had regions with solidified molten droplets and interconnected holes 10–100 µm in diameter, confirming that the material was submitted to very high temperatures and melted (Figure 2b). EDS analysis indicates the presence of uranium, oxygen, and carbon, similar to the pre-laser-heated sample. However, the number of orifices was greater than in the pre-laser-heated material, which is probably due to the release of carbon oxide gases from induced carbothermic reduction reactions. This assumption is also confirmed by the X-ray diffraction data obtained on the post-laser-heating samples, which showed a higher amount of UC_2−y_ phase when compared with the data obtained from materials before the laser heating (Figure 4b).

After laser heating, the Raman spectrum still showed two broad bands, centered at 1330 and 1580 cm^−1^ (Figure 5b), which compare well with the 1321 and 1596 cm^−1^ bands observed in the pre-laser-heated materials. However, a strong decrease in the intensity of the D-peak (resulting from defective and disordered graphite due to the breathing modes of sp^2^ atoms ring) was seen, pointing to a lower amount of the disordered phase in the laser-heated material. Moreover, the G-peaks of graphite were sharper than before heating, in line with a more crystallized sample.

Interestingly, the post-laser-heated material showed grain sizes in the range of 10–20 nm (Figure 7b), slightly above the dimensions observed before heating. This post-melting electron microscope analysis therefore confirms that the grain growth remained very limited, even during the melting–solidification process. Although grain-size-related melting point variations cannot be excluded in samples with smaller grains, the present investigation suggests that in the present samples, the grain size effect on the melting–solidification temperature was lower than the current experimental uncertainty. The still-nanometric size of the grains after laser heating suggests an effect of inhibiting their growth and coalescence by the excess of carbon, pointing to these materials as good candidates for spallation targets. 

## 4. Conclusions

Nanostructured, porous uranium dicarbide with crystal grain sizes ranging between 4 and 10 nm was studied as a promising candidate for spallation targets and nuclear fuel. The main objective of this work was to study the thermal stability, phase transitions, sintering, and grain growth of the nano-UC_x_ material. The compound was a U–C–O composite with mainly a UC_2_ phase. It consisted of irregularly shaped grains stacked over each other. The laser heating facility of JRC Karlsruhe (Germany), a suitable methodology for the study of high-temperature phase transitions in uranium carbides, was used in this work to study the effect of the small grain size on such phase transitions. 

The following conclusions can be drawn from the present investigation:

- The solidification point of nanostructured UC_2−y_ was observed to occur at 2713 K ± 30 K, and the α-UC_2_– β-UC_2_ solid–solid phase transition at 2038 K, both results being in line with existing literature data for the corresponding bulk material.
- Post-melting material characterization showed that the laser heating treatment up to melting induced grain growth even on the short time scale employed here (<500 ms), but grain sizes were still nanoscopic, ranging between 10 and 20 nm. Moreover, Raman spectroscopy showed that re-solidified grains contained less-disordered graphite compared to the as-fabricated ones.
- It can therefore be inferred that the excess carbon inhibited grain growth in the present samples.
- Although grain-size-related melting point variations cannot be excluded in samples with smaller grains, this investigation suggests that in the present samples, the grain size effect on the melting–solidification temperature was smaller than the current experimental uncertainty.
- Finally, the present study indicates that the nanostructured UC_2−y_ materials can be retained as good candidates for porous, nanostructured spallation targets in terms of their high-temperature resistance.

## Data Availability

Not applicable.
